# A computational screen for site selective A-to-I editing detects novel sites in neuron specific Hu proteins

**DOI:** 10.1186/1471-2105-11-6

**Published:** 2010-01-04

**Authors:** Mats Ensterö, Örjan Åkerborg, Daniel Lundin, Bei Wang, Terrence S Furey, Marie Öhman, Jens Lagergren

**Affiliations:** 1 Department of Molecular Biology and Functional Genomics, Stockholm University, Svante Arrheniusväg 20C, SE-10691 Stockholm, Sweden; 2 Stockholm Bioinformatics Centre (SBC), Stockholm University, Albanova, Roslagstullsbacken 35, SE-10691 Stockholm, Sweden; 3 School for Computer Science and Communication (CSC), Royal Institute of Technology (KTH), SE-10044 Stockholm, Sweden; 4 Institute for Genome Sciences and Policy (IGSP), Deptartment of Biostatistics and Bioinformatics; 5 Department of Computer Science, Duke University, 101 Science Dr, Box 3382, Durham, NC 27708, USA

## Abstract

**Background:**

Several bioinformatic approaches have previously been used to find novel sites of ADAR mediated A-to-I RNA editing in human. These studies have discovered thousands of genes that are hyper-edited in their non-coding intronic regions, especially in *alu *retrotransposable elements, but very few substrates that are site-selectively edited in coding regions. Known RNA edited substrates suggest, however, that site selective A-to-I editing is particularly important for normal brain development in mammals.

**Results:**

We have compiled a screen that enables the identification of new sites of site-selective editing, primarily in coding sequences. To avoid hyper-edited repeat regions, we applied our screen to the *alu*-free mouse genome. Focusing on the mouse also facilitated better experimental verification. To identify candidate sites of RNA editing, we first performed an explorative screen based on RNA structure and genomic sequence conservation. We further evaluated the results of the explorative screen by determining which transcripts were enriched for *A-G mismatches *between the genomic template and the expressed sequence since the editing product, inosine (I), is read as guanosine (G) by the translational machinery. For expressed sequences, we only considered coding regions to focus entirely on re-coding events. Lastly, we refined the results from the explorative screen using a novel scoring scheme based on characteristics for known A-to-I edited sites. The extent of editing in the final candidate genes was verified using total RNA from mouse brain and 454 sequencing.

**Conclusions:**

Using this method, we identified and confirmed efficient editing at one site in the Gabra3 gene. Editing was also verified at several other novel sites within candidates predicted to be edited. Five of these sites are situated in genes coding for the neuron-specific RNA binding proteins HuB and HuD.

## Background

The eukaryote cellular machinery has been shown to contain several alternative processing mechanisms acting on RNA. On the pre-mRNA level, alternative splicing is a well-known mechanism altering transcripts. This type of alternative processing is particularly important in the nervous system, where it helps determine the properties of many types of neurons [1]. Although RNA editing has received less attention, it is known to fine-tune messenger RNA composition by changing single nucleotides (nt). The most common enzymes to perform editing in mammals are the ADAR (*adenosine deaminase that acts on RNA*) proteins. The ADAR enzymes ADAR1 and ADAR2 convert adenosines to inosines (A-to-I) within double stranded RNA by a hydrolytic deamination (reviewed in [2]). Since inosine is interpreted as guanosine (G) by the splicing and translational machineries, ADAR editing effectively results in an A-to-G change that may alter the amino acid sequence encoded by the substrate. There are two types of A-to-I edited sites, (i) hyper-edited sites that are abundant in non-coding and untranslated regions of long, almost completely double stranded, stem loop structures [3,4] and (ii) selectively edited sites that consist of imperfect stem loop structures, often formed by an exon and a trailing intron sequence. To date, site selective editing has mainly been found in genes involved in neurotransmission.

The known substrates for site selective editing typically have a functional significance due to non-synonymous alteration of a codon that alters the amino acid sequence. Both RNA strands of a substrate stem often show high conservation of sequence as well as structure in species from human to chicken [5-7]. Imperfections in the form of bulges and internal mismatches are important structural features for site selective editing [8]. Even though only a handful of substrates have been identified, editing has proven to be important for the function of the developing brain in both invertebrates [9] and vertebrates [10-12].

The method we developed encompasses an initial explorative screen followed by a refinement of potential candidate sites using a novel scoring system. Our explorative screen for selectively edited sites consists of two components, the initial identification of candidate sites using RNA structure prediction and the subsequent evaluation of these sites using evolutionary sequence conservation. For the first, we developed the program *StemPrediction *to predict edited double stranded RNA stems within genomic transcripts that contain sequence pairs with approximate reverse complementarity. For the second, to specifically extract duplexes found in evolutionarily conserved regions, a novel conservation measure was developed and applied that employs multiple alignments of 17 vertebrate genomes [13]. In the refinement phase, we first used alignments of genomic data and an expressed sequence database [14] to target candidate sequences enriched for A-G mismatches between genomic and transcribed sequences. In addition, candidates were evaluated using a novel 6-bit scoring scheme based on characteristics for known A-to-I edited sites.

Similar ideas have been used previously to construct computational screens with the same purpose [3,15-17]. The hallmarks of these prior screens have been the A-G discrepancy and the clustering of adjacent discrepancies. Less used components involve conservation (usually mouse/human) and prediction of target RNA foldback structures. These studies have mainly led to the discovery of thousands of hyper-edited substrates where the editing events arise from inverted repetitive elements such as *alu *sequences. To avoid the detection of extensively edited long stem loop structures created by *alu *inverted repeats, we focused on the mouse genome that is devoid of these repetitive elements.

Our aim is to find single sites of selective editing that have the potential to re-code the open reading frame (ORF). To do this we use only coding sequences from well annotated mouse genes in order to focus on sequences destined for protein synthesis. Unique to our screen is also the scoring scheme based on features of known sites of selective editing. The result of applying our extended screen to the mouse genome gives a substantial number of novel putative substrates of which 45 have been experimentally tested. Of these, 38 derive from our combined explorative screen and refinement and an additional 7 candidates from the explorative screen alone. That is, in the latter 7 we looked for editing events within 7 highly conserved stem regions without requiring an A-G mismatch when comparing to the transcript database. Among the 38 candidates found in the combined screen and refinement, we identified the Gabra3 transcript. This gene that codes for the α3 subunit of the GABA_A _receptor has recently been found by us to be edited at one site, giving rise to an isoleucine to methionine change in the protein sequence. From sites identified using the explorative screen alone, we confirmed editing in several candidates, particularly in the neuron-specific RNA binding Hu-proteins. Our results imply that our method can be used to accurately identify novel substrates for site selective editing.

## Results

### Prediction of RNA stem structures within mouse genes

**Figure 1:**
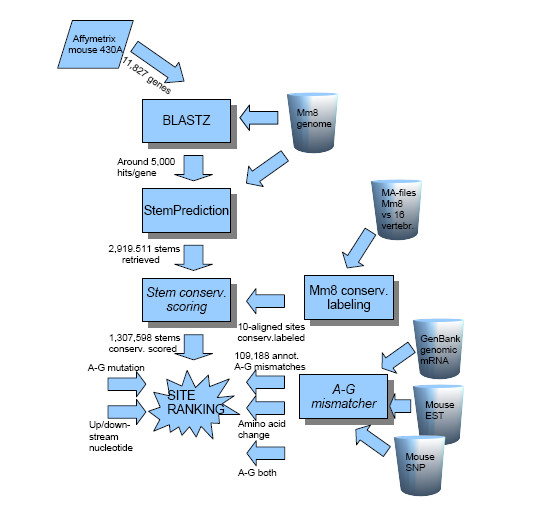
**Flowchart of the process**. The process of surveying and assigning potentially RNA A-to-I edited sites is here described.

Previous work in our laboratory have included the use of Mouse Genome 430A 2.0 Array (Affymetrix) to experimentally detect novel sites of A-to-I editing [18]. In this study, we use a novel bioinformatic method to search the sequences from these 11,827 well annotated mouse genes for new sites of editing in their open reading frames. An advantage of using the mouse genome instead of the human genome is the avoidance of Alu repeat elements known to be highly edited but so far without a well defined function. The mouse genome contains inverted repeats within transcribed regions having the potential of being double stranded RNA structures targeted for editing. Indeed, repeats are also edited in mouse with one documented example of editing in a SINE element of the CTN-RNA [19]. However, editing in the mouse repeats are much more infrequent, possibly due to the higher divergence of the different repetitive elements [20]. We first extracted the genome sequences corresponding to the genes on the above mentioned microarray from the mouse genome assembly release 8 (Mm8) [21]. BLASTZ [22] was then used to identify those transcribed sequences containing nearly exact reverse complementary pairs of subsequences, reasoning that these are likely to form RNA duplexes (Figure 1). Our BLASTZ search was restricted to mouse genomic regions that: (i) correspond to one of the 11,827 mouse genes; and (ii) are alignable with at least 10 other species in the multiple sequence alignments (MSA) consisting of the mouse genome aligned to 16 other vertebrates. The total number of sequence pairs extracted with BLASTZ was 53,729,218, an average of about 5,000 per gene.

### Identification of stems as potential substrates for ADAR enzymes

**Figure 2:**
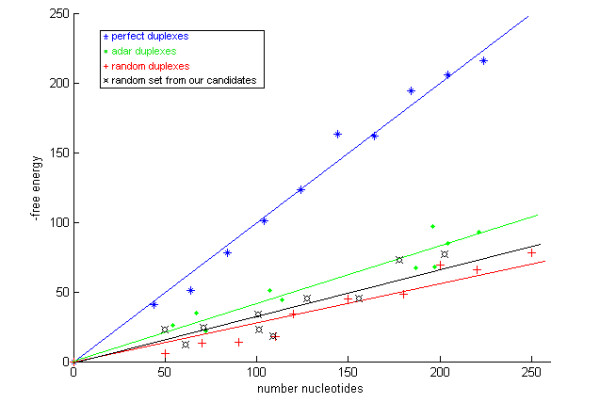
**Free energy as a function of duplex length**. The minimum free energy as a function of duplex length (in nt) for ten examples of different duplexes: perfect duplexes, known ADAR duplexes, random duplexes, and a random set from our candidate duplexes. We conclude that the trend is clear in the assumption that we would benefit from not being too strict in assigning parameters to StemPrediction.

**Figure 3:**
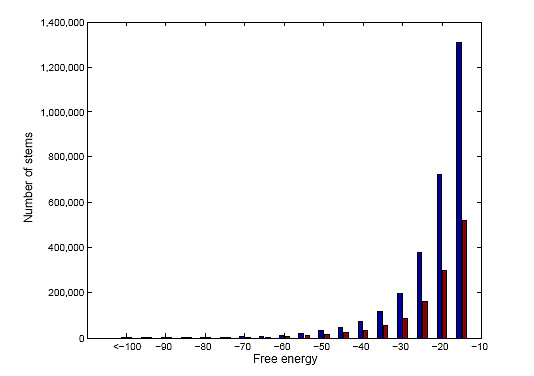
**The distribution of stems from StemPrediction**. Distribution of free energy for all the 2,919,511 stems retrieved from StemPrediction (blue bars), and the 1,307,598 stems having a predicted stem loop shorter than 10,000 nt (red bars).

Both sequence and structure are often phylogenetically well conserved at sites of selective editing. We used our novel program *StemPrediction *(see Methods) to filter the large sequence collection extracted above with BLASTZ for pairs of sequences exhibiting characteristics of known ADAR substrates (Figure 1). A key parameter was the *MAX_ENERGY *cut-off corresponding to minimum free energy for the stems. We avoided a strict cut-off, since the free energy for known ADAR substrates are often moderately low (Figure 2). On the other hand, an overly liberal cut-off would inevitably result in a vast amount of noise sequence pairs. Based on these considerations, *MAX_ENERGY *= -15 kcal/mol was chosen. When inspecting the results, we found it unlikely that a looser cut-off would yield any additional interesting predictions. The energy values for the retrieved stems ranged between the extremes -15 and -1,382. The empirical distribution is shown in Figure 3. The total number of retrieved stems was 2,919,511. Of those, 1,307,598 *candidate stems *with a maximum length of 5,000 nt were selected for further analyses.

### Identifying conserved stem structures

Using the multiple alignment of the mouse genome with several other genomes, we scored each candidate stem according to its level of conservation. Based on previously confirmed editing sites, we expected ADAR substrates to be highly conserved in terms of structure, at least in areas close to the edited site. Typically, it is the nucleotides in the helical regions of the ADAR substrates whose identity is conserved whereas nucleotides in non-helical regions are not, although their non-helical state is maintained. This is evident in a previous phylogenetic analysis that reveals an unusual sequence conservation within exonic and intronic sequences involved in RNA editing [5]. This turns out to also be true for editing substrates that consists of exon sequence entirely [23]. We therefore required a high conservation score on both stem arms of the putative substrates. In order to exclude regions of low conservation we defined the overall conservation score of a stem to be the score of its *lowest *scoring stem arm but the highest scored site on that arm (Table 1).

**Table 1:** Identified RNA stems

Conservation score	Sites	Gene overlapping sites	Areas	Gene overlapping areas	Stems
≥ 90	6,713	4,874	673	481	438
80-90	76,503	59,450	4,385	3,395	3,397
70-80	243,781	191,259	19,654	15,619	40,600
60-70	1,299,386	70,587	1,050,411	56,467	93,004
50-60	3,348,784	2,690,862	97,464	78,472	83,222
< 50	53,217,663	42,298,490	N/A	N/A	1,086,937

Total	58,192,830	45,315,522			1,307,598

Number of sites, gene overlapping sites (annotated gene that include any of the sites), areas (An area with conservation score *c *is a set of contiguous sites, with at least one site scoring *c *or higher, surrounded by 50 consecutive sites all having a score below *c*), gene overlapping areas (annotated gene overlapping any area, as defined), and number of predicted stems within the corresponding conservation score intervals are shown.

Using the mouse vs. 16 vertebrates multiple sequence alignment (MSA), we scored each site/nucleotide within the predicted stems according to its level of conservation (see Methods). This MSA attempted to align regions from the mouse genome to as many of the other 16 genomes as possible. Each mouse site included in an alignment containing at least 10 out of the 17 species was given a positive *conservation score *while all other positions were given a conservation score of zero. The conservation score for these *10-aligned *consists of two terms, a *parsimony term *and the *tree term*, both computed relative to a window of *k *nucleotides upstream and *k *nucleotides downstream of *s*. We found *k *= 10 to be suitable, i.e., the conservation score for *s *depends on the sites in a window of width 21 surrounding *s*.

The number of sites in the mouse genome that was given a positive conservation score was 58,192,830, approximately 2% of the mouse genome, and the values ranged from just above zero to 110 (Table 2). An *area *with conservation score *c *is a set of contiguous sites, with at least one site scoring *c *or higher, surrounded by 50 consecutive sites all having a score below *c*.

**Table 2:** The number of candidate stems in various conservation score intervals

Conserv. score	Stems	A-G mm
≤ 90	438	**51**
		**11.6%**

80-90	3397	**908**
		**26.7%**

70-80	40600	**10841**
		**26.7%**

60-70	93004	**20249**
		**21.7%**

50-60	83222	**14451**
		**17.3%**

< 50	1086937	**138285**
		**12.7%**

Total	1307598	**184785**
		**14.1%**

For each score is tabulated the total number of stems, the number of stems with A-G mismatch (typeset in bold), and the percentage of A-G mismatches (bold).

**Figure 4:**
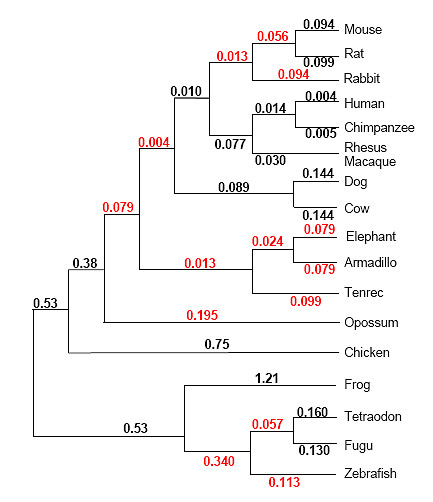
**Phylogenetic tree relating the 17 vertebrate species used to evaluate conservation**. Numbers on edges represent edge lengths measured in average substitutions per site. Black numbers are estimations made by Adam Siepel using PAML. Red numbers are estimated with the use of TimeTree [48] assuming local molecular clocks.

**Figure 5:**
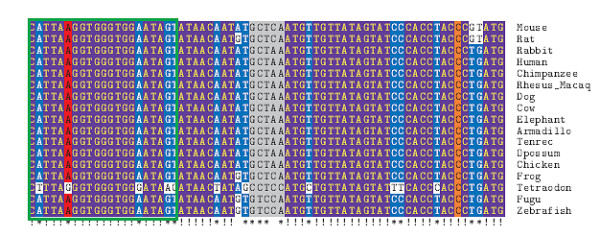
**Genomic alignment of species at the R/G site of *gluR-B***. A 17-species alignment, visualized with TeXshade [49], of the genomic region overlapping *gluR-B *at the R/G editing site. The column corresponding to the edited site is shown in red, while the complementary site is shown in orange. The loop is shown in grey. We note: (1) extreme conservation, (2) lost conservation in Tetraodon, (3) the A-G mutation occurring in Tetraodon in the edited column. The green rectangle surrounds a 21-column window used as an example in Methods.

**Figure 6:**
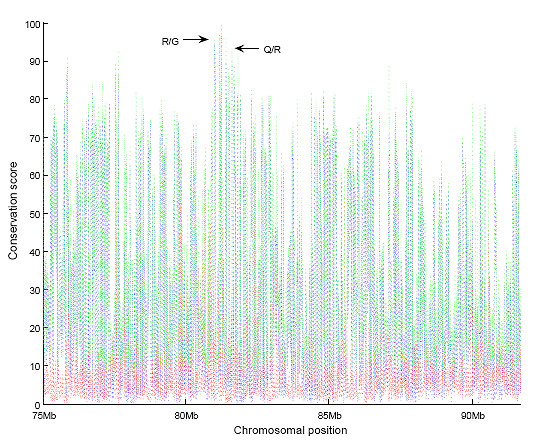
**The conservation score distribution for section 75-92 Mb of Mm8 chromosome 3**. The conservation score for a site (shown in green) is the sum of the parsimony term (red curve) and the tree term (blue curve) for that site. Approximate conservation score for the genome positions of GluR-B R/G (conservation score for highest scoring stem arm = 96.5) and GluR-B Q/R (93.4) are specified.

The idea behind using the parsimony term and the tree term is that the former should capture absolute conservation, i.e., its value will be high for sites in which very few mutations have occurred, while the latter should capture conservation in the mouse and human part of the tree which relates the aligned species (Figure 4). That is, a site in which several substitutions have occurred in some small subtree distant from mouse, but where no substitutions has occurred elsewhere, will have a high tree term value. As an example we used the alignment of a genomic sequence of the AMPA glutamate receptor, subunit B at the known R/G editing site (GluR-B R/G) (Figure 5). A boxed window (in green) of the first 21 nucleotides contains five substitutions altogether. All these have occurred in one species, resulting in a high tree term for this window. Further, a section of mouse chromosome 3 overlapping the positions for the editing substrates GluR-B Q/R and R/G, respectively, was plotted against the conservation score (Figure 6). From this graph we can conclude that the genome positions for these two substrates score higher than all other chromosomal positions.

### Identifying sites of editing

**Figure 7:**
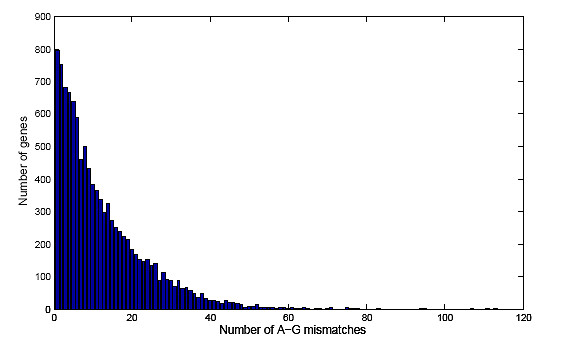
**Distribution of the number of genes that overlap a certain number of A-G mismatches**. The number of A-G mismatches are plotted against the number of genes in mouse. Bars for genes Ubc (which overlap 188 A-G mismatches), Mll5 (231), and Spna2 (420), are not shown.

To identify specific sites of selective A-to-I editing within the candidates selected by structure and phylogenetic conservation, a screen was made that discriminate between an A in genomic sequence and a G at the same position in EST data for an *individual*. This is typically an indication of an A-to-I editing site in the mRNA sequence. The genomic sequences used in the alignment were matched to the cDNA sequence, also called *genomic mRNA *below. However, the sequences in the databases correspond to many individuals, so an A-G mismatch may be caused by single nucleotide polymorphism (SNP). Therefore, we used the mouse SNP database to remove known SNPs of genomic origin from our A-to-G targets. However, it has previously been shown that over one hundred SNPs in human are most likely due to A-to-I editing [24,25]. Therefore, A-to-G SNPs verified by the sequencing of ESTs were not excluded from the screen. We used two databases, *mouse EST *[14] and *mouse SNP *[26], to extract the A-G mismatches. A total of 142,136 A-G mismatches were identified and of those 32,948 were rejected due to concurrent hits with a genomic origin in the SNP database. Thus, 109,188 high quality A-G mismatches were detected. The number of genes containing a certain number of A-G mismatches ranged between the extremes 0 and 420 according to the distribution in Figure 7. In 10,841 genes at least one A-G mismatch was detected.

For those regions assigned positive conservation scores, we evaluated our collection of candidate stems for A-G mismatch enrichment. We partitioned the spectrum of conservation scores into sections < 50, 50-60, 60-70, 70-80, 80-90 and ≥ 90. If conservation score and A-G mismatches both indeed are ADAR substrate characteristics, A-G mismatches will be enriched among candidate stems with high conservation scores. We evaluated this using a null hypothesis according to which an A-G mismatch is independent of A-to-I editing. Since we view: i) editing as the only possible explanation for dependence between A-G mismatch and conservation and ii) in order to get a computable p-value, we extend the null hypothesis to include independence between A-G mismatch and conservation. Absolute numbers and relative frequencies of A-G mismatches for various conservation scores are shown in Table 1. The frequency of A-G mismatch among stems with conservation score < 50 and 80-90 are 0.116 and 0.267, respectively (Table 2). The probability of having such a high discrepancy assuming that an A-G mismatch is a random phenomenon with the same distribution in both ranges of conservation score is < 10^-85^, this p-value was calculated with Hoeffding's bound [27]. We conclude that there is a correlation between editing and a high conservation score. In both ranges, some of the A-G mismatches could be attributed to random phenomena independent of editing, and we assume that the fraction of randomly occurring A-G mismatches is the same in both ranges. This fraction can be no larger than 0.116, indicating that the fraction of edited stems in the 80-90 range is at least 0.145, corresponding to 514 stems.

### Site ranking based on known substrates for site selective editing

To narrow down the number of candidates further, we utilized a site ranking scheme as a filter. We first imposed a minimum conservation score for a candidate to be evaluated by the site ranking scheme. Using the conservation scores for the predicted stems of known edited sites as a guide (Table 3), We set this cut-off conservation score to ≥ 75. Of the 53,729,218 sequences with complementary subsequences identified by BLASTZ, we found 2,524 stems with conservation scores above this cut-off. To further reduce the number of potential sites, we applied a site scoring criteria based on common features among known ADAR substrates. The known substrates are too few to apply a machine learning approach or to allow a good understanding of the relative importance of these criteria. So, our approach is purely heuristic. We used a bit-scoring scheme in which a candidate stem could have a maximum score of 6 (Table 4). The first two bits were used to credit conservation even further. To promote the conservation we decided to use the conservation score ≥ 80 and ≥ 90. The reason for this was that the 2 top scored known substrates regarding conservation (GluR-B: R/G 96 and Q/R 85) also are edited close to 100%. Assuming the editing frequency to be a quality marker for the conservation trait, we decided to add 2 bits in total for highly conserved stems, referred to as *cons_80 *and *cons_90 *in Table 4. The third bit specifically scores whether an A to G mutation is observed in the transcript data. This bit is called the *A-G mutation*. A stem has an A-G mutation if (i) it has an A-G mismatch when comparing genomic and transcribed sequences; and (ii) mouse and a closely related species have an A at the A-G mismatch site in the alignment, while species distant to the mouse have a permanent G at that position as shown for the GluR-B R/G site where Tetraodon has a genomic G at the R/G site (Figure 5). The fourth bit was used to reward distinct A-G mismatches in both stem arms since the probability of having A-G mismatches in both stem arms is significantly lower than the probability of having an A-G mismatch in only one stem arm. This bit is called *A-G_both*. To determine whether the A-G substitution would result in a change in the protein sequence, we downloaded all available mRNA and protein sequences from the Entrez gene site [28]. If amino acid changes appeared in the consensus protein sequence due to A-to-G changes it was scored as *annotated_aa_change*.

**Table 3:** Compilation of known ADAR substrates with respect to how they are captured by the pipe.

substrate			A-G mismatcher^a^			
**name**	**Entrez gene**	**Codon change**	**Mm**	**Hs**	**stemPrediction^*b*^**	**stemConservation^*c*^**

Adar2	Adarb1	intron	n/a	n/a	yes	no
Bc10	Blcap	Y/C	yes	yes	yes	no
	Cyfip2	K/E	yes	yes	yes	no
	Flna	Q/R	no	yes	yes	no
	Ednrb	Q/R	n/a^*d*^	No^*e*^	yes	no
Bc10	Blcap	Y/C	yes	yes	yes	no
GluR-B	Gria2	Q/R	yes	yes	yes	yes
		R/G	yes	yes	yes	yes
GluR-C	Gria3	R/G	no	yes	yes	yes
GluR-D	Gria4	R/G	yes	yes	yes	yes
GluR-5	Grik1	Q/R	no	yes	yes	no
GluR-6	Grik2	Q/R	no	no	yes	no
		Y/C	no	no	yes	yes
		I/V	no	no	yes	yes
5-ht2c	Htr2c	I/V_1	no	no	yes	yes
		I/V_2	no	no	yes	yes
		N/S	no	no	yes	yes
	Igfbp7	R/G	yes	yes	yes	no
		K/R	yes	yes	yes	no
	Kcna1	I/V	yes	yes	yes	yes

^*a *^States whether an A-G mismatch has been found in mouse (Mm) and human (Hs).

^*b *^States whether Stemprediction has assigned any stem overlapping an edited position, regardless of the stem ranking (or if it is the correct one).

^*c *^States whether the stem according to column 4 has a conservation score ≥ 75.

^*d *^To our knowledge this site has not been annotated in mouse which is also emphasized by low sequence similarity between the 2 species.

^*e *^A-G mismatcher does not detect the annotated site but finds 2 additional A-G mismatches in the vicinity, inferring an I/M and a D/G codon change, respectively.

**Table 4:** Filters used in the candidate scoring process

Site score	Description
Cons 80	The predicted stem has a conservation score of ≥ 80
Cons 90	The predicted stem has a conservation score of ≥ 90
AG mutation	If a distant sub-tree has a DNA coded G at the position of an A-G mismatch (see also Figure 5).
AG_both	There are A-G mismatches on both stem arms
Annotated aa change	The A-G mismatch results in an amino-acid discrepancy
ds G	The nucleotide downstream of the A-G mismatch position is a G

It has previously been shown that there is a sequence bias in the vicinity of an edited adenosine [29]. Hence, we used algorithms for calculating information content [30] to sort out if and how to score a nearest neighbor distribution of an edited site (see Methods). The information content in this case is related to whether there is a pattern of nucleotide disposition that differs from the expected with respect to the background distribution. This means that if there is a background distribution of equal amounts of the four different nucleotides and we also find a distribution of equal amounts of nucleotides at a position, we can gain no information at that position, i.e., 0 bits. The calculations are based on known selectively edited sites in mammals where the literature reports more than 40% editing. Consequently 24 sequences were used to calculate the information pattern ± 200 nt around an edited position. Expectedly, we find the highest degree of information just adjacent to the targeted adenosine. The upstream and downstream neighbor had 0.43 and 0.50 bits respectively. The downstream preference of a guanosine (0.27 bit) and the higher total information content motivated us to score downstream guanosines of a candidate editing site, (*ds_G*). We compiled a list of 38 selected candidates having a site score of ≥ 3 (Table 5). In this list, Gabra3, appeared with a score of 4. Using other methods this substrate has subsequently been shown by us to be highly edited at one site (I/M) in mouse brain [7].

**Table 5:** A-to-I editing candidates

Gene	Codon change	Cons 80	Cons 90	AG mutation	AG both	Annotated aa change	ds G	Total sum
Adipor1	K:R	1	0	1	1	0	1	4
Ccnc	Q:R	1	1	0	1	0	1	4
Elavl1	S:G	1	0	1	1	0	1	4
Gabra3	I:M	1	0	0	1	1	1	4
Gabarapl2	syn	1	0	1	1	0	1	4
Cnot2	N:S	1	1	1	1	0	0	4
Tra1	syn	1	0	1	1	0	1	4
Acin1	K:R	1	0	1	1	0	1	4
Eif4a2	syn	1	0	1	1	0	1	4
Eif4e2	K:R	0	0	0	1	1	1	3
Ptpra	Q:R	1	0	0	1	0	1	3
Etv3	Q:R	1	0	0	1	0	1	3
GluR-B	syn	1	1	0	0	0	1	3
GluR-B	I:V	1	1	1	0	0	0	3
Lmo4	K:R	1	0	0	1	0	1	3
Elavl2	K:R	1	0	0	1	0	1	3
Elavl2	syn	1	0	1	1	0	0	3
Stk22c	Q:R	1	0	0	1	0	1	3
Dhx15	Q:R	1	0	0	1	0	1	3
Fzd1	S:G	1	0	1	0	0	1	3
Ywhag	K:R	1	0	0	1	0	1	3
Ptn	S:G	0	0	1	1	0	1	3
Arfip2	Q:R	0	0	1	1	0	1	3
Tial1	M:V	1	0	1	1	0	1	3
Gabarapl2	S:G	1	0	0	1	0	1	3
Crsp6	S:G	0	0	0	1	1	1	3
Ets1	syn	1	0	1	1	0	0	3
Atp5b	Q:R	1	0	0	1	0	1	3
Cnot2	M:V	1	1	0	1	0	0	3
Cnot2	Q:R	1	1	0	1	0	0	3
Cnot2	K:E	1	1	0	1	0	0	3
Tra1	K:R	1	0	0	1	0	1	3
Tra1	S:G	1	0	0	1	0	1	3
Nmt1	K:R	1	0	0	1	0	1	3
Sox9	K:R	1	0	0	1	0	1	3
Sox9	syn	1	0	0	1	0	1	3
Akt1	R:G	1	0	0	1	0	1	3
Evl	S:G	1	0	0	0	1	1	3
Kns2	N:D	1	0	1	1	0	0	3
Pcbp2	Q:R	1	0	0	1	0	1	3
Ap2m1	K:R	1	0	0	1	0	1	3
Ap2m1	Q:R	1	0	0	1	0	1	3
Actr1a	Q:R	1	0	0	1	0	1	3
Pten	Q:R	1	0	0	1	0	1	3
Hnrph2	Q:R	1	0	0	1	0	1	3
Hnrph2	K:R	1	0	0	1	0	1	3
Timm8a	K:R	1	0	0	1	0	1	3
Ube1x	N:S	1	0	0	1	1	0	3

The final list of candidates (47) which are chosen from all sites having a score ≥ 3 (124).

### Verification of editing using high throughput sequencing technology

To validate editing in the 38 candidates, we used the amplicon *454 sequencing technology*. The advantage of using this technique is that even low levels of editing can be detected with high accuracy. The collection of 454 sequences retrieved for each of the 38 candidate genes were aligned to the mouse genome. The number of sequences aligning to a candidate gene, and thus the number of alignment rows, ranged from 46 to 1,267. The 454 output contains a *phred score *for each position indicating the risk of erroneous sequencing for the position in question. That is, even though one can give a general estimate for 454 sequencing errors, the *phred score *provides a much better estimate for any given specific position. In most cases the phred score was reported to be between 20 and 30 corresponding to 1% and 0.1% risk respectively. Furthermore, all alignments showing any sign of poor quality were discarded. A total of 175 positions were found where a genomic A was replaced by a G in at least one of the sequences. The Gabra3 transcript was found to be edited 93% of the time in these analyses (Table 6). Another gene found to be edited was Elavl2 (also known as mHuB). The alignments corresponding to this site contained 625 sequences out of which 15 (2.4%) showed an A-G replacement at one site (Table 6). This editing event causes an amino acid change from aspargine to aspartic acid (N/D). Also another site confirmed to be edited within this transcript causes an isoleucine to valine (I/V) change in 1.4% of the transcripts. The mHuB protein is a neuron-specific RNA binding protein with 3 RNA recognition motifs (RRMs). Both of the edited sites are situated in RRM3. In addition, another 8 sites where the editing frequency was higher than 0.6% were found, 6 of these leading to amino acid changes (Table 6). Among these was Elavl4 (also known as HuD), another neuron specific RNA binding protein.

**Table 6:** Verified novel sites of editing

Gene	Coverage	#G	#A	Freq. of G	aa change
Gabra3	679	631	48	92.93%	I/M
Elavl2	633	9	624	1.422%	I/V
Elavl2	625	15	610	2.400%	N/D
Elavl4	443	4	439	0.903%	3' UTR
Elavl4	462	3	459	0.649%	3' UTR
Elavl4	220	2	218	0.909%	T/A
Matr3	220	3	217	1.364%	R/G
Stk22c	209	2	207	0.957%	D/G
Ube1x	349	3	346	0.860%	I/M
Xpo7	390	3	387	0.769%	D/G
Hnrph2	265	2	263	0.755%	K/E

Top edited genes with sites having editing p-value < 0.0001 (combined phred score for the probability of erroneous sequencing). Coverage indicates the total number of sequences, #G is edited sequences and #A the number of non-edited sequences, giving the frequency of editing (Freq. of G) and the implied amino acid change (aa change).

## Discussion

We have described an explorative screen for selectively A-to-I edited sites, based on two components, RNA stem structure and conservation of the corresponding sequence. For the stem structure, we use a free energy threshold and characteristics of known ADAR substrates while the conservation score is used to rank stems. Unlike previous attempt to detect sites of editing we have focused on modifications within encoded sequence [3,15-17]. However, in a recent genome wide screen a fundamentally different approach to detect novel sites of selective editing was used where repetitive elements where filtered [31]. In line with our results Li et al. stress the finding of widespread editing at low levels (< 2%) and few edited sites that give rise to a change of the translational code.

An assay was designed for our explorative screen that tests whether highly conserved stems are enriched for positions with an A-G mismatch between the genomic and the transcribed sequence. The result of the evaluation is that A-G mismatches are significantly enriched in highly ranked stems. Comparing stems in the 80-90 conservation score range with those in the < 50 range yields an estimate of 514 edited stems in the former. Given that true editing events are located in highly conserved stems we consider the frequency of A-G mismatches in the < 50 range to be the background we see in the other conservation ranges. Consequently a fraction of 0.116 could always be expected to be noise associated with each of the other ranges. Therefore 514 (908 - 0.116 × 3397) stems in the range 80-90 are believed to be stems wherein true editing occurs. The same type of comparison between each of the three intervals 70-80, 60-70, and 50-60 and the interval < 50 yields an estimate of 18,074 edited stems in the combined conservation score range 50-80. These values are surprisingly high given the number of currently known ADAR substrates. However, it is noticeable that the conservation score range ≥ 90 contains relatively few stems with an A-G mismatch. We find two possible explanations for this: (i) several of the known ADAR substrates are in this range but have been excluded in order to not bias the calculations with bona-fide substrates, (ii) the editing efficiency is lower than 50% and not registered as an A to G change in the database and (iii) known functional edited sites often have a G in fish and amphibians that are more distantly related to mammals and this prevents a very high conservation score. Thus, a conservation score of a true editing event is often impaired by the fact that species far from human/mouse often have a template DNA G at the editing site, which lowers the conservation score.

We refined our screen by including several additional components of which A-G mismatch between genomic and transcribed sequences is one. Our refinement was applied to mouse orthologs of the known human ADAR substrates. As seen in Table 3, of the known selectively edited sites, 4 are contained in a stem structure that: (i) has an A-G mismatch in mouse as well as human, (ii) has a free energy below the threshold and (iii) has a conservation score above 75. By restricting ourselves to structures with conservation score above 75, we lose some of the known ADAR substrates but the majority satisfy this requirement. From the final list, it is worth noting that the R/G and Q/R sites of GluR-B, and the I/V site of Kcna1 are among the absolute top ranking candidates (Table 7). This is a strong indication that our screen in total has an intrinsic capacity to detect ADAR targets. Further, we found one novel substrate for site selective editing to be highly scored. The Gabra3 transcript coding for the α3 subunit of the GABA_A _receptor got a score of 4. This transcript was verified to be edited to 93% using amplicon 454 sequencing on RNA extracted from the mouse brain (Table 6 and [32]. The site of editing in Gabra3 gives rise to an amino acid codon change from isoleucine to methionine (I/M) within exon 9 [23]. Thus, the high score in the present computational screen indicates that it is possible to detect novel sites of selective editing using this method. Noteworthy is that in previous attempts to find substrates for editing, the Gabra3 transcript was not detected. The unique feature of our screen compared to others is that we limit our analysis to encoded sequence in combination with RNA secondary structure conservation and hallmarks for efficient site selective editing. Out of the final list, 45 candidates were investigated further. By using the 454 sequencing method the sequence of several hundred to a thousand single transcripts can be analyzed and thereby the accuracy in editing efficiency determination is extremely high. Altogether, we found editing in 175 positions where the A-G discrepancy could not be explained by either sequencing or alignment errors. The top 11 sites (including Gabra3) are listed in Table 6. Noteworthy is that five of these come from our explorative screen alone. That is, we detect signs of true editing without the A-G mismatch requirement and exclusively due to extreme conservation traits of our predicted stems. Out of those, three sites are located in the ORF of neuronal Hu proteins B and D (also known as Elavl, embryonic lethal abnormal vision (Drosophila)-like). The Hu family members HuB and HuD (Elavl 2 and 4) play important roles in neuronal differentiation and proliferation [33]. They consist of three RNA binding domains (RRMs) and have been shown to be involved in RNA processing events that regulate expression of NF1 (Neurofibromatosis type 1) [34], Ikaros [35] and CGRP (Calcitonin gene-related peptide) [36] in neuronal cells. Also, plasticity in SNP composition in these genes have been implied in Parkinson disease [37]. Interestingly, in HuD the T/A editing site (indicating the amino acid change) is situated in RRM 2, and in HuB both the I/V and the N/D editing sites are in RRM 3. Even though only low levels of editing were detected in HuB and HuD, one should keep in mind that samples from total brain was used. It is therefore possible that editing in these genes is higher in certain regions of the brain and that it is of importance to achieve a tissue specific regulation of alternative splicing. Moreover, since these are neural specific genes, increased levels of editing in non-neuronal tissues might be a way to down regulate these proteins. Although further analyses are required, our list of substrates with a low level of editing gives a hint of cell specific gene regulation by RNA editing, With one recent exception [31], this type of low level editing has previously not been possible to detect using other methods.

**Table 7:** Scores of known editing substrates

Gene	Codon change	Cons 80	Cons 90	AG mutation	AG both	Annotated aa change	ds G	Total sum
GluR-B	R:G	1	1	1	0	1	1	5
GluR-B	Q:R	1	0	1	0	1	1	4
Kcna1	I:V	1	1	0	0	1	0	3
Kcna1	syn	1	0	0	1	0	1	3
Cyfip2	Q:R	1	0	0	1	0	1	3

## Conclusions

Using our explorative screen in combination with 454 sequencing, it is possible to find novel sites of editing within coding sequence at levels that have previously not been possible. Our findings also point to a risk in basing an entire screen for A-to-I edited sites on A-G discrepancies between genomic and cDNA sequences annotated in the database, since many of the candidates here found to be edited came from the explorative screen alone.

## Methods

### BLASTZ and StemPrediction

We used NCBI gene ID:s to download a complete set of genbank files for the 11,827 unique genes represented on the Affymetrix 430A microarray. Genes that could not be unambiguously mapped to a Genbank entry were discarded. We used BLAT [38] to align the head and tail sequences (100 nt of the 5' and 3'-end of a gene, respectively) to the corresponding chromosome. All sequences that could not be completely and uniquely aligned to their corresponding chromosome (NCBI build 36) were also discarded. BLAT was used with default parameters with the exception of *MIN_IDENTITY*. *MIN_IDENTITY *= 100 was chosen since we wanted to eliminate incomplete alignments. To determine potential stem loop forming structures, first BLASTZ [22] was used to align each sequence to the reverse complement of itself, using parameter settings as shown in Table 8. We constructed a custom weight matrix for these alignments that reflects the contribution of each base pairing to the stability of the structure including the non-standard G-U pairing (G-T in DNA sequence) (Table 9). Resulting alignments were further filtered using our *StemPrediction *software. *StemPrediction *first determines the lowest energy confirmation of a stem loop structure formed by the BLASTZ aligned sequences using RNAfold [39]. Parameter settings, see Table 8, allow potential stem loops to be further filtered based on characteristics of the predicted structure such as the RNAfold determined minimum free energy, the length of the stem, and the number of paired and unpaired nucleotides (bulges) in the stem. Stems from disjoint structures can be joined to create larger structures if stems sequences are within a specified distance of each other. These characteristics of stem loop structures have been previously shown to be important in RNA editing [40-42].

**Table 8:** Parameters used with BLASTZ and StemPrediction.

	Parameter	Value	Description
BLASTZ	O	150	Gap
	E	100	Gap
	K	500	Maximal segment pair (MSP) score
	L	500	Gapped alignment threshold
	W	6	Word size

StemPrediction			
	MIN_ARM_LENGTH	16	Minimum stem arm length (nt)
	MAX_ENERGY	-15.0	Minimum free energy of the stem
	MAX_BULGE_SIZE	5	Maximum number of unpaired nt on a single strand in the stem
	MAX_BULGE_BASES	7	Maximum number of unpaired nt on both strand in the stem
	MAX_GLUE_DISTANCE	10	Maximum distance for two stems to be glued (joined) StemPrediction
	MAX_FILTER_ENERGY	-15.0	Minimum free energy of the glued stem

**Table 9:** Weight matrix used with BLASTZ.

	A	C	G	T
A	80	-100	-100	-100
C	-100	120	-100	-100
G	20	-100	120	-100
T	-100	20	-100	80

### A-G mismatch idenitification

The most recent set of 11,827 well annotated gene sequences, including UTR's, and exon coordinate annotations for all transcript isoforms, were downloaded [43]. The coordinates gave a complete set of genomic coding sequences. We used two databases as of February 2007, a mouse *EST database *[14] and an *SNP database*, build 126 [26]. The genomic mRNA sequences were aligned to the EST database using BLASTN [44] in order to deduce A-G mismatches between the template DNA and the expressed sequences. To reduce the risk of promoting an A-G mismatch originating from sequencing errors and/or low quality alignments, we discarded alignments shorter than 100 nt and alignments containing ≥ 20 mismatches. We further used the SNP database to remove A-G mismatches likely to have a polymorphic genomic origin.

### Mouse genome sequence conservation labeling

The Mm8 version of the mouse assembly was used. Each genomic site included in cross-species alignments that contained at least 10 of the 17 species was scored according to:

The parsimony term for column *s *is calculated as the negative logarithm of the p-value for the parsimony score in the window centered at *s*. The details of how the p-value is computed are found in [13] where this algorithm is entitled *parsimony-based method for MCS detection*. The calculation is done with respect to the structure of the species tree (Figure 4), the tree's edge lengths, and a substitution rate matrix (we follow [13] and use the HKY neutral substitution rate matrix [45]). When calculating the tree term we consider all columns in the window simultaneously and we observe where in the tree nucleotides deviating from the consensus are found:

*n *= total #leaves

*m *= # leaves in subtree with mutations

*d*_*i *_= number of mutations in column i

*k *= total number of columns

This value will be large if all deviating nucleotides are isolated to some small subtree (c.f. the GluR-B example shown in Figure 5 where in the boxed window all deviations are found in Tetraodon). In this case the parsimony term will be lowered by the five columns having a substitution but the tree term will be rather high since they are all in the same one-species subtree.

### Site scoring scheme

A scoring scheme containing bits cons_80, cons_90, A-G_mutation, A-G_both, annotated_aa_change and ds_G were used. The values of bits cons_80, and cons_90 was retrieved directly from the mouse genome conservation labeling output. The A-G_mutation, and A-G_both was similarly retrieved directly from the A-G mismatches output correlated with the mouse genome conservation labeling and *StemPrediction *respectively. In scoring annotated_AA_change, we aligned amino acid sequences for a gene with the translated genomic mRNA using DIALIGN [46]. The amino acid sequences were retrieved from NCBI *Entrez gene *[28], either protein sequences from the *Entrez protein *or translated sequences from *Entrez nucleotide*. If a position annotated as an A-G mismatch also showed a corresponding amino acid discrepancy, this site was scored (bit annotated_aa_change). To compile sequence biases around an edited site (i.e. bit ds_G) we calculated the information content ± 200 nt from a selected set of 24 edited adenosines from the known substrates.

Where *H(l) *is the uncertainty (entropy) [47] at position *l*, *n *is the 4 nt to be summed over, and *f(n, l) *is the frequency of nucleotide *n *at *l*. The total information at position *l *is: *I(l) *= 2 - *H(l)*. From the information calculation we decided to bit score a downstream G.

### 454 amplicon sequencing

RNA was isolated from mouse brains using TRIzol (Invitrogen). For the first-strand cDNA synthesis random primers was used. PCR was carried out with primers specific for known edited regions. Fused to the primers were adaptor oligonucleotides specific for the following sequencing procedure. Superscript III RT (Invitrogen) was used in all reverse transcription reaction, and FastStart High Fidelity PCR System (Roche) was used in all PCR reactions. To exclude that the samples were contaminated with genomic DNA, reactions in absense of RT enzyme were also carried out. Amplified PCR products were run on a 1.5% agarose gel and the bands were cut out and gel purified. All amplified PCR products were pooled. In the 454 procedure, the PCR products were immobilised on DNA capture beads. The bead DNA was emulsified in a water-in-oil mix that contains reagents for amplification. Hence, one bead corresponds to one fragment or transcript. The amplified fragments were loaded onto a PicoTiterPlate™- one bead/well = one read. The plate was then subjected to sequencing reagents using the pyro-sequencing technique (Roche).

In Table 6 we collect the top candidates where the risk of erroneous sequencing is less than 0.0001. This probability is calculated using the phred scores. A phred score of 25 indicates the probability of a sequencing error is 10^-2.5^. Since we manually discarded A-G discrepancies due to poor alignments, we assume that the probability of falsely assigning an A-G mismatch as a true editing event to be solely dependent on the phred score (sequencing error). In 40 out of 175 cases, the p-value was found to be < 0.0001. In addition to Gabra3, the 10 sites showing highest editing frequency are listed in Table 6.

## Authors' contributions

JL and MÖ initiated the project and outlined the general ideas. ME and ÖÅ built the explorative screen with some help from DL. ME and ÖÅ performed all the data analysis. TSF and BW developed the program *StemPrediction*. All authors read and approved the final manuscript.
